# The Correlation of Carpal Tunnel Pressure with Clinical Outcomes following Ultrasonographically-Guided Percutaneous Carpal Tunnel Release

**DOI:** 10.3390/jpm12071045

**Published:** 2022-06-27

**Authors:** Jui-Chien Wang, Chung-Yi Li, Po-Yen Ko, Tung-Tai Wu, Kuo-Chen Wu, Fong-Chin Su, I-Ming Jou, Po-Ting Wu

**Affiliations:** 1 School of Medicine, College of Medicine, National Cheng Kung University, Tainan 701401, Taiwan; mercer8840up@gmail.com; 2 Department of Public Health, Collage of Medicine, National Cheng Kung University, Tainan 701401, Taiwan; cyli99@mail.ncku.edu.tw; 3 Department of Public Health, College of Public Health, China Medical University, Taichung 404328, Taiwan; 4 Department of Healthcare Administration, College of Medical and Health Science, Asia University, Taichung 413305, Taiwan; 5 Department of Orthopedics, National Cheng Kung University Hospital, College of Medicine, National Cheng Kung University, Tainan 704302, Taiwan; madseason329@hotmail.com; 6 Department of Biomedical Engineering, National Cheng Kung University, Tainan 701401, Taiwan; uhuhuwu@gmail.com (T.-T.W.); fcsu@ncku.edu.tw (F.-C.S.); 7 GEG Orthopedics Clinic, Tainan 701002, Taiwan; 8 Department of Orthopedics, Kuo’s General Municipal Hospital, Tainan 700002, Taiwan; nike0421@so-net.net.tw; 9 Medical Device Innovation Center, National Cheng Kung University, Tainan 701401, Taiwan; 10 Department of Orthopedics, E-Da Hospital, Kaohsiung 824005, Taiwan; 11 School of Medicine, College of Medicine, I-Shou University, Kaohsiung 824410, Taiwan; 12 Department of Orthopedics, College of Medicine, National Cheng Kung University, Tainan 701401, Taiwan; 13 Department of Biochemistry and Molecular Biology, College of Medicine, National Cheng Kung University, Tainan 701401, Taiwan

**Keywords:** carpal tunnel syndrome, treatment outcome, carpal tunnel pressure, surgery, ultrasound

## Abstract

Background: To evaluate the correlation between carpal tunnel pressure (CTP) and the clinical presentations, and to explore the possible predictors for the postoperative recovery pattern in patients with carpal tunnel syndrome (CTS). Materials and Methods: Consecutive patients with idiopathic CTS following percutaneous ultrasound-guided carpal tunnel release (UCTR) were enrolled. CTP was measured preoperatively and immediately after operation. The Boston Carpal Tunnel Questionnaire (BCTQ) and the cross-sectional area (CSA) of median nerve were recorded preoperatively and at 1, 3, and 12 months postoperatively. Results: 37 patients (37 hands; 8 men and 29 females; median age, 59.0 years) were enrolled. CTP significantly decreased immediately from 40.0 (28.0–58.0) to 13.0 (8.0–20.0) mmHg after UCTR. BCTQ scores significantly improved at 1 month postoperatively, and the improvement trend persisted until 12 months postoperatively (*p* < 0.001). Preoperative CTP was positively correlated with preoperative CSA and preoperative BCTQ scores (*p* < 0.05, all). Using group-based trajectory modeling, all patients were categorized into the “gradual recovery” or “fast recovery” group. Higher preoperative CTP was significantly associated with a faster recovery pattern (odds ratio: 1.32). Conclusions: Preoperative CTP was well correlated with the clinical presentations and might be a useful predictor for the postoperative clinical recovery pattern.

## 1. Introduction

Carpal tunnel syndrome (CTS) is recognized as one of the most common compression neuropathies and work-related disorders in the upper extremities worldwide. The risk factors contributing to CTS include repetitive movements, prolonged tasks [1], awkward postures of the wrist joint [2,3], and prolonged loading of the wrist joint on the palmar side [4]. In the pathophysiology of CTS, elevated CTP is believed to be a possible pathomechanism leading to CTS [5,6].

The correlation between various clinical parameters and the pre-/post-operative Boston Carpal Tunnel Syndrome Questionnaire (BCTQ) scores, representing symptom severity, has been widely discussed in the literature. The investigated clinical parameters included the cross-sectional area (CSA) of median nerve, the severity of nerve conduction study (NCS), gender, and age, and some of them have been individually reported to be correlated with the perioperative BCTQ scores or the postoperative improvement of the BCTQ scores in various studies [7,8,9,10,11,12,13,14]. However, to date, there is still no consensus on which clinical parameters are significantly correlated to clinical outcomes.

Furthermore, there is a paucity of studies investigating the correlation between the CTP and the severity of symptoms. Lee et al. [7] reported that the CTP was correlated with the clinical signs (e.g., two-point discrimination) and the duration of symptoms before carpal tunnel release (CTR). In contrast, Ahn et al. [15] reported that the CTP was not correlated to the NCS grade or the CSA of the median nerve. To date, the correlation between the CTP and clinical outcomes is still unknown.

Therefore, we reported the perioperative changes of the CTP and clinical outcomes in patients with CTS undergoing percutaneous ultrasound-guided carpal tunnel release (UCTR). We aimed to evaluate the correlation between the CTP and the clinical presentations, and the possible predictors for the postoperative clinical recovery pattern. In the present study, we hypothesized that the preoperative CTP is correlated to the preoperative clinical presentations and might be a predictor for postoperative clinical recovery pattern.

## 2. Materials and Methods

### 2.1. Ethics Statement

The human study was approved by the Institutional Review Board of the senior author’s hospital (No. A-ER-110-255), and all methods were performed in accordance with the approved guidelines.

### 2.2. Participants

This was a case-control study. From May 2013 to March 2015, consecutive patients with idiopathic CTS undergoing percutaneous UCTR were enrolled. The inclusion criteria were as follows: clinical symptoms and signs (pain, numbness, tingling, and waking at night), and corresponding results of NCS in the median nerve. The exclusion criteria included peripheral neuropathy, diabetes, previous upper extremities or wrist surgeries, inflammatory joint disease, cervical disorder, or previous carpal tunnel release. The electrophysiological diagnosis of CTS using NCS was established in accordance with the guidelines of the American Association of Electrodiagnostic Medicine [16]. According to the results of NCS, the severity of CTS was classified as mild, moderate, and severe [16]. Furthermore, the values of sensory nerve action potential (SNAP) and compound motor action potential (SMAP) were recorded for further analyses.

### 2.3. The CTP Measurement and Percutaneous UCTR

**Figure 1:**
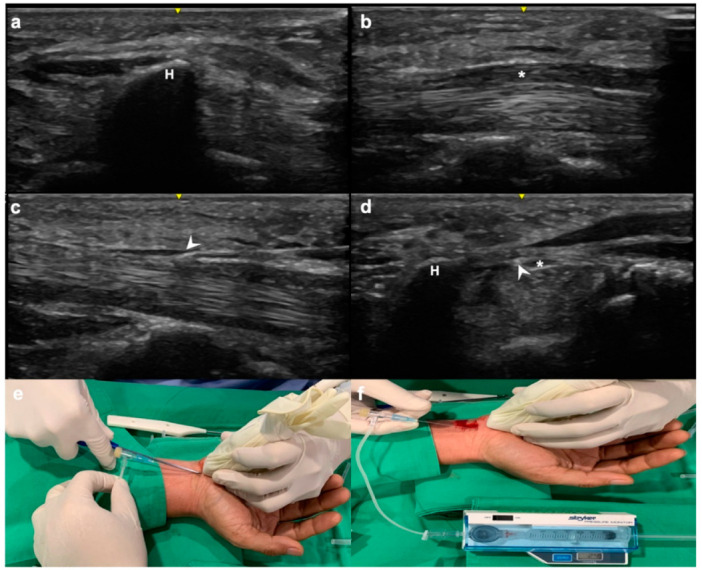
The step-by-step procedure of the carpal tunnel pressure (CTP) measurement and carpal tunnel release (CTR) under ultrasonographical guidance. First, the median nerve, transverse carpal ligament, and hamate hook were quickly examined using ultrasound (US). The hamate hook was aimed at the center of the region of interest (ROI) in the sagittal view (**a**). The transducer was transversely moved towards the radial side until the full view of the median nerve (**b**), and then moved back a little toward the ulnar side to make ROI next to the median nerve. The needle of the CTP measurement gauge was percutaneously inserted into the carpal tunnel, and the tip of the needle was placed in the center of ROI in the sagittal view to align the hamate hook in the transverse direction (**c**). The transducer was turned transversely to make sure again that the tip of the CTP measurement gauge was next to the median nerve, within the carpal tunnel, and at the same level as the hamate hook (**d**). The percutaneous CTR was done under ultrasonographic guidance (**e**). After the surgical release, the CTP was measured again and recorded (**f**). H, hamate hook; asterisk, median nerve; arrowhead, the tip of the needle of the CTP measurement gauge.

The CTP was measured before and after an operation via the pressure measurement system [17] (Intra-Compartmental Pressure Monitor System, 295-1 Pressure Monitor, Stryker^®^ Surgical, Kalamazoo, MI, USA). The affected hand and forearm were placed in the supination posture, and the wrist was placed in the neutral position on the operating table. The sonographic linear-array transducer (5–10 MHz, SonoSite, Inc., Bothell, WA, USA) with 10 MHz was enclosed in a sterile surgical glove and was put at the center of the midcarpal joint of the wrist. After a sterile preparation and well-localization of the median nerve, flexor tendons, transverse carpal ligament, and the useful landmarks, the hamate hook was centered in the region of interest (ROI) under the sagittal view (Figure 1a). Then the ultrasound (US) transducer was transversely moved towards the radial side until full view of the median nerve was obtained (Figure 1b). The transducer was moved back a little towards the ulnar side to keep the ROI next to the median nerve, and the needle of the CTP measurement gauge was inserted into the carpal tunnel through a puncture wound after injection of local anesthesia over the puncture wound. Meanwhile, the tip of the needle of the CTP measurement gauge was placed in the center of ROI in the sagittal view to align the hamate hook in the transverse direction (Figure 1c). Then, the transducer was turned transversely to make sure again that the tip of the CTP measurement gauge was next to the median nerve, within the carpal tunnel, and at the same level as the hamate hook (Figure 1d). After the preoperative CTP measurement, the entire transverse carpal ligament was released by an ultrasound-guided operation under local anesthesia, similar to our previous study [18,19,20] (Figure 1e). After percutaneous UCTR surgery was performed, the postoperative CTP was immediately measured again (Figure 1f). During the whole procedure, the local anesthesia was given only over the subcutaneous layer. There was no application of a tourniquet.

### 2.4. The CSA of Median Nerve and Clinical Outcomes

The CSA of median nerve was measured at the carpal inlet using an ultrasound system by an orthopedic surgeon with experience of musculoskeletal sonography and percutaneous UCTR for more than five years. The data acquired was analyzed by the ImageJ software (National Institutes of Health, Bethesda, MD, USA). The ultrasound was positioned at the level of the scaphoid and the pisiform, which was approximately localized at the level of the distal wrist crease. The CSA of median nerve was calculated by an elliptical area method. The measurements were repeated at the same examination session and the results were averaged. The intraclass correlation coefficient of the two measurements was 0.90. The clinical outcomes were assessed using the BCTQ score, including symptom severity scale (BCTQ-S) and functional status scale (BCTQ-F), by an independent observer blinded to the patient information [21,22]. The CSA of the median nerve and the BCTQ scores were recorded before surgery and at one month, three months, and twelve months postoperatively.

### 2.5. Trajectory Groups of Postoperative Clinical Recovery Pattern

Group-based trajectory modeling (GBTM) was aimed at detecting heterogenous subpopulations and has been often applied in previous studies [23,24,25]. In the present study, GBTM was utilized to identify the postoperative clinical recovery pattern of the BCTQ-Total scores. The GBTM method assumes several latent groups of distinct polynomial functions of repeated measurements, namely trajectories. The optimal number of trajectory groups and the appropriate order of trajectories were determined by considering clinical plausibility and several statistical criteria: (1) the Bayesian information criterion (BIC) was minimal [26]. (2) the average of the posterior probability of group membership was >0.7; (3) odds of correct classification was >5; and (4) the estimated group probabilities and the proportions of the sample assigned to the group were reasonably close to each other; and (5) the confidence intervals for group membership probabilities are reasonably tight [27,28].

### 2.6. Statistical Analysis

Due to the relatively small sample size of our patients, non-parametric tests were used to perform the statistical analyses if the normal distribution of data was not assumed. Values of baseline characteristics were presented as the median (interquartile range) or number (percentage). The differences in various continuous outcome variables between pre- and post-operation at various points in time during the follow-up were assessed using the Wilcoxon Signed-rank test. The test for trends in changes of various outcome variables over time was exanimated using linear regression models with the GEE method, which accounts for correlated data. Spearman rank correlation coefficients was used to determine the strength and direction of associations between variables, and the correlation between preoperative CSA and CTP was assessed using Pearson correlation coefficient because the data were considered to be normally distributed by the Kolmogorov–Smirnov test.

To identify the potential predictors for different groups of trajectory, the comparisons of different baseline parameters between trajectory groups were performed using the Mann–Whitney U test for continuous variables and the Fisher’s exact test for the proportion. Only the preoperative CTP was included in the analysis due to its high correlation with the postoperative CTP. Then, binary logistic regression was used to estimate the crude and covariate adjusted odds ratios (ORs) and 95% confidence intervals (CIs) of certain trajectory groups in relation to selected baseline variables and covariates. The level of significance was set at *p* < 0.05. The data were analyzed using the SPSS statistical package (version 17.0; SPSS, Chicago, IL, USA).

## 3. Results

### 3.1. Demographic Data

The thirty-seven hands of the thirty-seven CTS patients (20 right hands and 17 left hands) were enrolled in this study. There were eight males and 29 females with a median age of 59.0 (50.5–66.5) years. The median symptom duration was 7.0 (6.0–12.0) months. Based on the NCS grade, there were 11 patients categorized into mild grade, six patients into moderate grade, and 20 patients into severe grade. The detailed information about the preoperative CTP, SNAP, and CMAP in each NCS severity grade are listed in Supplementary Table S1.

### 3.2. Outcomes after Percutaneous UCTR

The CTP significantly and immediately decreased from 40.0 (28.0–58.0) to 13.0 (8.0–20.0) mmHg following percutaneous UCTR in our patients (*p* < 0.05, Table 1). For postoperative symptom severity and functional outcomes, there were significant improvements in the BCTQ-S/BCTQ-F/BCTQ-Total scores at one month after operation (*p* < 0.001) compared with preoperative status, and the improvement trends were significant until 12 months postoperatively (*p* < 0.001). With respect to the CSA of the median nerve, there was no significant decrease in values postoperatively until 3 months after operation (*p* < 0.05, Table 1).

### 3.3. Correlation between the Preoperative CTP and the Clinical Presentations

The preoperative CTP was significantly and positively correlated with the preoperative CSA of the median nerve and the preoperative BCTQ-S/ BCTQF/BCTQ-Total scores (*p* < 0.05, Table 2). The preoperative CTP was moderately correlated with the BCTQ-S, BCTQ-F, and BCTQ-Total scores. However, no significant correlation was noted between the preoperative CTP and the duration of symptoms, the severity grade of NCS, SNAP, and CMAP.

### 3.4. Postoperative Improvement Trajectory

Based on the GBTM, it was found that the two-group trajectory classification had the smallest BIC score (−131.03), with both groups following a cubic trend. In addition, the average posterior probability for the two groups was the same at 0.998, and the odds of correct classification were 168.0 and 1797.0, respectively, which indicated that both trajectories reasonably matched the empirically determined groups.

**Figure 2:**
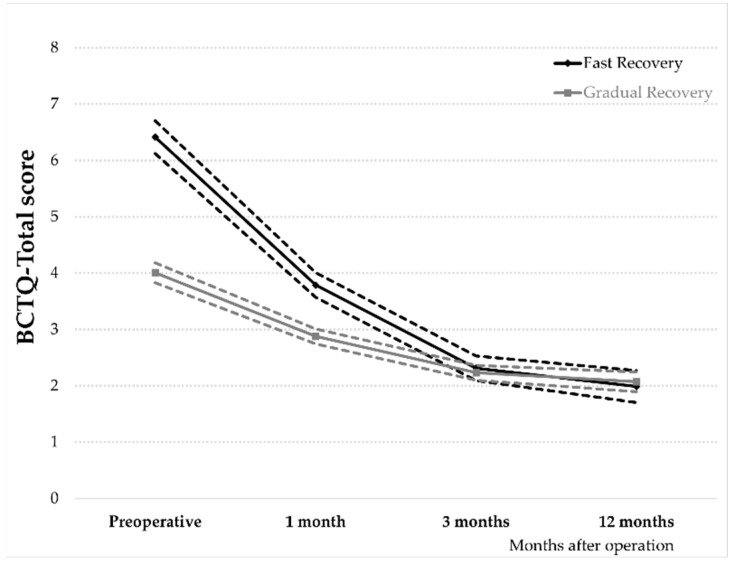
The trajectories and 95% confidence intervals of the perioperative recovery pattern of the Boston Carpal Tunnel Questionnaire (BCTQ)-Total scores after percutaneous ultrasound-guided carpal tunnel release.

The 37 patients enrolled were categorized into two different trajectories of postoperative BCTQ-Total scores (Figure 2), namely, (i) Gradual recovery pattern (72.85%) including 27 cases, and (ii) Fast recovery pattern (27.15%) including 10 cases. The group with a fast recovery pattern converged to a similar level as the gradual recovery group in the 3rd month after operation.

### 3.5. Predictors for Trajectory Pattern

Various baseline variables were compared between the two trajectory groups (Table 3). There were significant differences in both the preoperative and postoperative CTP between the two trajectory groups (*p* < 0.05). There were no significant differences in age, gender, the affected side, the preoperative CSA of the median nerve, and the NCS data, including SNAP, CMAP, and the severity grade between the two groups. The “Fast recovery” group was associated with significantly higher pre-/post-operative CTP, as compared to the “Gradual recovery” group. Because the pre- and post-operative CTP were highly correlated with each other (R = 0.7328, *p* < 0.0001), we included only the preoperative CTP in the binary logistic regression model to quantify the association between the preoperative CTP and the trajectory patterns of the BCTQ-Total scores. After adjusting for age and sex, the patients (hands) with higher preoperative CTP tended to have significantly higher odds of experiencing the “Fast recovery pattern” of the BCTQ-Total scores (Table 4). An mmHg increase in CTP was associated with a 32% increase in the likelihood of experiencing the “Fast recovery pattern” of the BCTQ-Total scores. (Adjusted odds ratio (OR) 1.32, 95% CI: 1.05–1.66, *p* < 0.05).

## 4. Discussion

In the present study, after operation, the CTP immediately and significantly decreased below the threshold reported to do damage to the median nerve (30 mmHg) [29]. The preoperative CTP had a significant and positive correlation with the preoperative CSA of the median nerve, and the preoperative BCTQ-S/BCTQ-F/BCTQ-Total scores. All the patients enrolled were categorized into the two-group trajectories by GBTM, “Gradual recovery” and “Fast recovery” patterns, respectively, based on the distinctive characteristics of the postoperative clinical recovery pattern of the BCTQ-Total scores. The higher preoperative CTP led to a faster recovery pattern with a covariate adjusted odds ratio of 1.32. This implied that the preoperative CTP was well correlated with the preoperative clinical presentations and might be a predictor of the postoperative clinical recovery pattern.

CTP has been proposed as one of factors to cause CTS and is related to the pathological process of CTS [5,6]. The mean CTP in a normal population has been reported from 8 to 16 mmHg [17]. The elevated CTP would damage the median nerve when the increase in CTP is over 30 mmHg [29]. It was reported in previous studies [7,15,17], using either open or endoscopic approaches, that the measured CTP significantly decreased immediately after carpal tunnel release (CTR). In the present study, the application of percutaneous UCTR also showed a significant decrease in the CTP postoperatively, and the significant improvement in the BCTQ-Total scores postoperatively was also shown in our results. It was then speculated that percutaneous UCTR could be considered as an effective alternative for operation, as indicated by previous reports [19,30,31,32]. Several kinds of methods and equipment have been reported in the application of CTP measurement [7,17,33]. The measured CTP value could also be affected by the measurement area in the carpal tunnel [7,15] and the wrist posture such as flexion, extension, or neutral position [7,15,17]. To date, there is no consensus on the standard measurement protocol of the CTP. In the present study, the measured area of the CTP is over the level of the hamate hook with the wrist in neutral position, similar to that reported by Uchiyama et al. [33].

The correlation between the CTP and the clinical presentations still remains uncertain. The preoperative CTP had been reported to be significantly correlated to 2-point discrimination (2-PD) scores and the duration of symptoms [7], but not correlated to the NCS grade or the CSA of median nerve [15]. To the best of our knowledge, there have not been any studies investigating the correlation between the CTP and the BCTQ scores. In the present study, the preoperative CTP was shown to be significantly correlated with the preoperative CSA of the median nerve and the BCTQ-S/BCTQ-F/BCTQ-Total scores, but not with the duration of symptoms, the NCS data including SNAP, CMAP, and the severity grade. Furthermore, Kim et al. [9] reported that the preoperative CSA of the median nerve was significantly correlated with the preoperative BCTQ-S and BCTQ-F scores. In our results, the preoperative BCTQ-Total scores were fairly correlated with the preoperative CSA of the median nerve (Supplementary Table S2) and moderately correlated with the preoperative CTP. Therefore, we speculated that the preoperative CTP reflected the clinical presentations, corresponding to the current pathophysiology hypothesis that the CTP is one of the factors leading to CTS.

To investigate the postoperative clinical recovery pattern following percutaneous UCTR, GBTM was utilized to identify different postoperative clinical recovery patterns of the BCTQ-Total scores. Patients in the present study could be categorized into two groups, “Gradual recovery” group and “Fast recovery” group, respectively. We found that the preoperative CTP was a significant predictor for the postoperative clinical recovery pattern of the BCTQ-Total scores following percutaneous UCTR. The higher preoperative CTP significantly corresponded to a faster recovery pattern of the BCTQ-Total scores postoperatively. To the best of our knowledge, this is the first study reporting an association between the preoperative CTP and the postoperative clinical recovery pattern. The baseline BCTQ scores have been reported to be positively correlated with the improvement of the BCTQ scores at six months after operation, indicating that it is among the most important factors in predicting symptom relief after surgical release [34]. In the present study, the utilization of GBTM could provide a resolution to analyze the dynamic trajectory pattern of symptom improvement [24]. We found that the preoperative CTP was moderately correlated with the preoperative BCTQ scores, and the higher preoperative CTP led to a faster recovery pattern with a covariate adjusted odds ratio of 1.32. Clinically, various NCS severity classifications do not always accurately reflect the severity of CTS [35]. The Martin-Gruber anastomosis, the normal anatomical innervation variant, might lead to an underestimation of CTS severity in NCS [36]. Furthermore, postoperative recovery after CTR has been reported to be independent of the electrodiagnostic study severity [14]. Our results also indicated that the NCS results is not related to the postoperative recovery pattern.

There were some limitations to our study. First, the patient number in the study was small, even the differences in our aims are significant. Theoretically, higher CTP might lead to more severe damage to the median nerve that is presented with worse NCS results, including severity grade, SNAP, and CMAP. In the current study, our results failed to demonstrate the correlation between NCS parameters and the preoperative CTP. In addition to the individual variations of CTP in symptomatic CTS, the limited patient number is another possible reason leading to the insignificant results. Thus, a larger scale study is necessary to confirm our findings. Second, up to date, there is no consensus on the standard measurement protocol of the CTP and no specific instrument for the CTP measurement. However, the measured CTP significantly decreased after percutaneous UCTR and was significantly correlated with the clinical presentations. Our results indicated the measured pressure could represent the real CTP.

## 5. Conclusions

The present study noted an immediate decrease in the CTP following percutaneous UCTR. In addition, the preoperative CTP was positively correlated with the CSA of the median nerve and the BCTQ-S/BCTQ-F/BCTQ-Total scores. Patients with a higher preoperative CTP were more likely to experience a faster recovery pattern of the BCTQ-Total scores. Our results indicated that the preoperative CTP was well correlated with various clinical presentations over the postoperative stages and might be a useful predictor for the postoperative clinical recovery pattern.

## Figures and Tables

**Table 1:** Clinical outcomes before and after percutaneous ultrasound-guided carpal tunnel release.

Parameters	Preoperative	Postoperative	1 Month	3 Month	12 Month	β-Coefficient	*p* for Trend
CTP (mmHg)	40.0 (28.0–58.0)	13.0 (8.0–20.0) ^#^	N.A.	N.A.	N.A.	N.A.	N.A.
CSA (mm^2^)	13.0 (11.0–14.0)	N.A.	12.0 (12.0–14.0)	12.0 (10.0–12.0) ^#^	10.0 (10.0–12.0) ^#^	−0.24	0.028
BCTQ-Total	4.4 (3.6–5.8)	N.A.	2.8 (2.6–3.5) ^#^	2.2 (2.0–2.3) ^#^	2.0 (2.0–2.0) ^#^	−0.87	<0.001
BCTQ-S	2.2 (1.8–2.7)	N.A.	1.5 (1.3–1.8) ^#^	1.1 (1.0–1.2) ^#^	1.0 (1.0–1.0) ^#^	−0.42	<0.001
BCTQ-F	2.3 (1.8–2.8)	N.A.	1.5 (1.3–1.8) ^#^	1.0 (1.0–1.2) ^#^	1.0 (1.0–1.0) ^#^	−0.45	<0.001

Values are presented as the median (interquartile range); *p* value, examination of time trend of the study group using linear regression models with the GEE method; β-coefficient, regression coefficient indicating time trend; **^#^** The significant between-timepoint difference in comparison with the preoperative status using the Wilcoxon Signed-rank test; CSA: Cross-sectional area of median nerve; BCTQ: Boston Carpal Tunnel Questionnaire; BCTQ-S: Boston Carpal Tunnel Questionnaire-symptom severity scale; BCTQ-F: Boston Carpal Tunnel Questionnaire-functional status scale.

**Table 2:** Correlation between the preoperative carpal tunnel pressure (CTP) and the clinical presentations.

Parameters	Preoperative CTP	
	r	*p*-Value
Duration of symptoms (month)	0.310	0.084
CSA of median nerve (mm^2^)	0.444	0.011
Nerve conduction study		
SNAP	−0.212	0.245
CMAP	−0.077	0.677
Severity grade	0.222	0.222
BCTQ-Total	0.685	<0.001
BCTQ-S	0.561	0.001
BCTQ-F	0.738	<0.001

Spearman rank correlation was used to determine the correlation between variables. Pearson correlation coefficient was used to determine the correlation between the CSA of the median nerve and the preoperative CTP because data were considered to be normally distributed by the Kolmogorov-Smirnov test. SNAP, sensory nerve action potential; CMAP, compound motor action potential.

**Table 3:** Comparisons of clinical characteristics of patients between gradual and fast recovery groups.

Characteristics	Group 1 (Gradual Recovery; N = 27)	Group 2 (Fast Recovery; N = 10)	*p*-Value
Preoperative CTP (mmHg)	35.0 (25.0–40.0)	59.0 (55.0–70.0)	<0.001
Postoperative CTP (mmHg)	11.0 (6.0–16.0)	24.0 (17.0–40.0)	0.001
Age (years)	59.0 (51.0–71.0)	59.0 (48.0–65.0)	0.355
Gender (male)	7 (25.9%)	1 (10.0%)	0.404
Affected Side (Right)	14 (51.9%)	6 (60.0%)	0.725
Preoperative CSA of median nerve (mm^2^)	12.0 (11.0–14.0)	14.0 (13.0–16.0)	0.112
Nerve conduction study			
SNAP	10.7 (6.8–21.9)	7.8 (5.2–13.2)	0.296
CMAP	5.9 (3.5–8.3)	5.7 (3.3–7.2)	0.932
Severity grade			0.214
Mild	10 (37.0%)	1 (10.0%)	
Moderate	3 (11.1%)	3 (30.0%)	
Severe	14 (51.9%)	6 (60.0%)	

Values are presented as the median (interquartile range) or number (percentage). The between-group differences were analyzed using the Mann–Whitney u test for continuous variables and the Fisher’s exact test for the proportion; CTP, carpal tunnel pressure; CSA, cross-sectional area; SNAP, sensory nerve action potential; CMAP, compound motor action potential.

**Table 4:** Odds ratio of fast recovery group assignment in relation to baseline variables.

	Crude Odds Ratio (95% CI)	*p*-Value	Adjusted Odds Ratio (95% CI)	*p*-Value
Preoperative CTP	1.20 (1.05–1.36)	0.005	1.32 (1.05–1.66)	0.018
Age (years)	0.96 (0.90–1.02)	0.166	0.91 (0.79–1.04)	0.167
Gender (male)	0.32 (0.03–2.98)	0.315	0.02 (<0.01–2.76)	0.117

The association between clinical characteristics (the preoperative CTP, age, and gender) and the postoperative recovery pattern of the BCTQ-Total scores were analyzed using binary logistic regression. The results were presented as crude and adjusted odds ratio, respectively.

## Data Availability

The datasets used during the current study are available from the corresponding author on reasonable request.

## Supplementary Materials

The following supporting information can be downloaded at: https://www.mdpi.com/article/10.3390/jpm12071045/s1, Table S1: The preoperative carpal tunnel pressure (CTP) and nerve conduction study (NCS) data in different severity grades; Table S2: The correlation between the cross-sectional area (CSA) of median nerve and the Boston Carpal Tunnel Questionnaire (BCTQ) scores before and after percutaneous ultrasound-guided carpal tunnel release (UCTR).
