# Supplementary material for: The Correlation of Carpal Tunnel Pressure with Clinical Outcomes following Ultrasonographically-Guided Percutaneous Carpal Tunnel Release
Source: J Pers Med. 2022 Jun 27;12(7):1045. doi: 10.3390/jpm12071045 (PMC9325166; doi:10.3390/jpm12071045)
Supplement: Supplementary file 1 [file jpm-12-01045-s001.zip › jpm-1768953-supplementary.pdf]

Table S1. The preoperative carpal tunnel pressure (CTP) and nerve conduction study (NCS) data in different severity grades.

NCS grade	Preoperative CTP (mmHg)	SNAP (μV)	CMAP (mV)
Total	40.0 (28.0, 58.0)	10.4 (6.5, 16.9)	5.9 (3.5, 7.6)
Mild (n=11)	29.0 (22.0, 51.0)	16.8 (10.3, 24.3)	8.3 (6.8, 8.7)
Moderate (n=6)	51.0 (36.0, 60.0)	12.0 (8.0, 17.0)[#]	8.0 (5.9, 10.4)[#]
Severe (n=20)	40.0 (33.0, 58.0)	6.7 (4.0, 10.7)[#]	3.8 (2.8, 5.3)[#]
p-value	0.295	0.007	<0.001

Values are presented as the median (*interquartile range*).

p-value, based on the Kruskal-Wallis test for the global test of difference among the three groups.

[#]Significant between-group differences in moderate and severe groups using the the Mann-Whitney U test as the post hoc test.

Table S2. The correlation between the cross-sectional area (CSA) of median nerve and the Boston Carpal Tunnel Questionnaire (BCTQ) scores before and after percutaneous ultrasound-guided carpal tunnel release (UCTR)

	BCTQ							
**The CSA of median nerve**	Preoperative		Postoperative					
			1 Month		3 Month		12 Month	
	r	p-value	*r*	p-value	*r*	p-value	*r*	p-value
Preoperative	0.350	0.034						
1 Month			0.042	0.804				
3 Month					-0.171	0.312		
12 Month							0.246	0.142

Spearman rank correlation analysis was used to determine the correlation.
